# Importance of neonatal immunoglobulin transfer for hippocampal development and behaviour in the newborn pig

**DOI:** 10.1371/journal.pone.0180002

**Published:** 2017-06-28

**Authors:** Kateryna Goncharova, Liudmyla Lozinska, Ester Arevalo Sureda, Jarosław Woliński, Björn Weström, Stefan Pierzynowski

**Affiliations:** 1 Department of Biology, Lund University, Lund, Sweden; 2 R&D Anara AB, Trelleborg, Sweden; 3 Department of Animal Physiology, The Kielanowski Institute of Animal Physiology and Nutrition, Polish Academy of Sciences, Jabłonna, Poland; 4 Department of Medical Biology, Institute of Rural Health, Lublin, Poland; INIA, SPAIN

## Abstract

Neurological disorders are among the main clinical problems affecting preterm children and often result in the development of communication and learning disabilities later in life. Several factors are of importance for brain development, however the role of immunoglobulins (passive immunity transfer) has not yet been investigated. Piglets are born agammaglobulinemic, as a result of the lack of transfer of maternal immunoglobulins *in utero*, thus, they serve as an ideal model to mimic the condition of immunoglobulin deficiency in preterm infants. Thirty six, unsuckled newborn piglets were fed an infant formula or colostrum and supplemented orally or intravenously with either species-specific or foreign immunoglobulin and then compared to both newborn and sow-reared piglets. Two days after the piglets were born behavioural tests (novel recognition and olfactory discrimination of conspecifics scent) were performed, after which the piglets were sacrificed and blood, cerebrospinal fluid and hippocampi samples were collected for analyses. Both parameters of neuronal plasticity (neuronal maturation and synapse-associated proteins) and behavioural test parameters appeared to be improved by the appearance of species-specific porcine immunoglulin in the circulation and cerebrospinal fluid of the piglets. In conclusion, we postulate possible positive clinical effects following intravenous infusion of human immunoglobulin in terms of neuronal plasticity and cognitive function in preterm infants born with low blood immunoglobulin levels.

## Introduction

Neurological disorders as well as behavioral and neuropsychiatric problems are among the main developmental problems in preterm children which can result in the development of communication and learning disabilities later in life [1]. The pathophysiology leading to the neurodevelopmental problems of premature infants and children is complex [2]. There is increasing evidence that both developing neurons and oligodendrocytes are at risk in the premature brain [3], with the potential to cause gross structural damage or more subtle disruptions in measures of connectivity [4,5,6]. Children born preterm are more likely to have white matter brain abnormalities early on resulting in a higher risk of cognitive dysfunction [7].

In humans, maternal antibody transfer via the placenta to the fetus starts as early as the 13^th^ week of gestation. However, the level of immunoglobulin G (IgG) in the fetal circulation is relatively low (5–10% of the maternal level) between weeks 17–22, reaching 50% of the maternal level by week 32 and usually exceeding the maternal plasma level at delivery [8]. However, since more and more children are born prematurely, some even before the 28^th^ week of gestation, the transfer of passive immunity is often insufficient and preterm babies display lower levels of total IgG [9,10], a subject which is often overlooked.

Immunoglubulin (IG) preparations for intravenous infusion are broadly used in clinical practice to treat a variety of sicknesses, as they are able to modulate various immunological pathways at both the humoral and cellular levels, including inflammation, antigen presentation, cell growth and apoptosis [11]. Exogenous IGs demonstrate high efficacy in the treatment of sepsis and acute lung injury in adults [12,13]. Intravenous infusion of IGs is on the FDA list of drugs for the treatment of *myastenia gravis* and showed positive neuroprotective effects for patients with Alzheimer disease during phase I and II clinical trials [14,15]. Since preterm neonates are at increased risk of developing life-threatening infections as well as neurological problems, the potential advantages of exogenous IG administration could be even greater than that observed in adults. However, the mechanisms of action of IGs, as well as their possible importance for cognitive development in newborns has yet to be investigated.

Recent studies from our lab [16,17] have highlighted the influence of IG on brain development, behavior and survival in preterm and newborn piglets, which are naturally hypogammaglobulinemic at birth. The “extra-immunological” effects of IG on central nervous system development are poorly understood and the treatment of preterm, symptomatically “healthy” infants, with IG in order to ensure their proper development is still not included in common healthcare guidelines. Piglets, like all other ungulates, are born without any passive immunity and the transfer of IG from the gut (following consumption of the mother’s colostrum) to the bloodstream of the newborn piglets takes place within the first 24 hours of life. The newborn pig model which is accepted as a relevant model for human neonates [17,18,19], especially for babies born prematurely, could also be used to study the impact of immunoglobulins on brain development in human neonatology.

The main aim of our study was to investigate whether supplementation of IG to newborn, healthy piglets could support brain development in terms of cognitive function and the improvement of neuronal plasticity.

## Materials and methods

All experimental procedures were approved by the Malmö-Lund regional Ethics Review Committee on Animal Experiments (approval № M142-14). All efforts were made to minimize animals’ suffering.

### Animals

The experiment was carried out on cross-bred ((Yorkshire x Swedish Landrace) x Hampshire) pigs (*Sus scrofa domesticus*) obtained from a specific pathogen-free herd (Vindfälle 810, 268 68 Röstånga, Sweden). A total of 36 piglets, 18 males and 18 females, from 10 litters, from multiparous sows, born at fullterm, were used in the study. The piglets were selected for the study provided they weighed between 1–1.5 kg and that they were of the correct gender (in order to allow us to have equal numbers of male and female piglets in the study). Immeadiately after birth the piglets were removed from the sow and placed in a straw bedded area, under a heating lamp (150 W) until they were transported (within 2,5 hours following birth) to the departmental animal facilities. All piglets were individually numbered and their body weight was measured both at the beginning and at the end of the experiments. During the experiments the piglets were housed together in their groups, in a clean, dry, stable area/crate with a dry towel for bedding, at a temperature of between 28–30°C.

### Experimental design

The thirty six piglets were randomized into 6 groups with equal numbers of male and female piglets in each group. Group IF (n = 6) was fed an infant formula (Similac Special care 24, Abbott Nutrition, Columbus, Ohio, USA). Group BC (n = 6) was fed bovine colostrum containing 109.16 mg IgG/ml. Group IF+IGLD (n = 6) was fed the infant formula and given an intravenous (i.v.) infusion of swine serum—representing a low dose of swine immunoglobulins. Group IF+IGHD (n = 6) was fed the infant formula and given an i.v. infusion of purified porcine immunoglobulin—representing a high dose of swine immunoglobulins. Group SC (n = 6) was fed swine colostrum containing 100.88 mg IgG/ml. At the start of the experiment (immediately after arriving to the animal facility and initial blood sampling), the IF + IGLD and the IF + IGHD groups of piglets were infused, via the umbilical vein, with either a sterile preparation of normal swine serum containing 25.22 mg IgG/ml (16 ml/ kg b.wt, Group IF+IGLD) or porcine immunoglobulin containing 66.96 mg IgG/ml (23 ml/kg b.wt, Group IF+IGHD), respectively. All piglets were fed their respective diets via a stomach tube in a volume of 10 ml/kg body weight, every two hours for up to 12 hours (six feedings). After this the piglets were fed exclusively with the infant formula every two hours for up to 48 hours (to the end of experiment).

In addition, unsuckled newborn piglets (Group NB, n = 6) that were not subjected to any form of treatment, were sacrificed within 1 h after birth and included as controls.

### Diets and immunoglobulin preparation

Swine and bovine colostrum were hand milked from 10 sows during farrowing and five cows after calving. Colostrum was pooled and stored fresh in aliquots of about 500 ml at 4°C during the experiment. Ten ml of the colostrum pool was centrifuged at 20,000 x *g* for 60 min at 4°C, the lipid layer at the top and the bottom pellet were removed, and the remaining colostrum whey was then stored at—20°C until use.

The normal swine serum was prepared from the blood obtained from three sows, which was centrifuged, pooled, sterile filtrated and stored at -20°C until use. The porcine immunoglobulin preparation was obtained from the pool of blood plasma obtained from three multiparous sows, by ammonium sulphate precipitation [20]. The IG preparation was sterile filtrated and stored at -20°C until use. The total amounts of immunoglobulin administered to the piglets in each experimental group, either via i.v. injection or orally, are presented in Table 1.

**Table 1:** Dose of respective immunoglobulins administered to piglets in experiment.

Dose, mg/kg b.wt.						
Group	NB	IF	BC	IF+IGLD	IF+IGHD	SC
Swine IG, mg/kg b. wt.	-	-	-	404	1540	6053
Bovine IG, mg/kg b. wt.	-	-	6550	-	-	-

Unsuckled newborn piglets (NB, n = 6) and newborn piglets fed with either an infant formula (IF, n = 6), bovine colostrum (BC, n = 6), an infant formula + i.v. infusion of sow serum (IF+IGLD, n = 6), an infant formula + i.v. infusion of porcine immunoglobulins (IF+IGHD, n = 6), or swine colostrum (SC, n = 6).

### Behavioural tests

The piglets’ behaviour was estimated 48 hours after being born using two different tests. An overhead video camera was used to record the piglets in the behavioural tests, which took place in a testing arena (120 cm×150 cm) with grey plastic walls and a rubber floor.

To assess the short-term memory and specific exploratory behaviour of the piglets, the *novel object recognition* test *(NOR)* was performed [21,22]. This test consisted of a 3 minute exploration phase and a 3 minute test phase, which were separated by a 4 minute inter-phase interval. During the exploration phase each pig was placed into the arena with two unfamiliar objects; objects were chosen based on previous findings in pigs [22,23,24]. For the inter-phase interval, the pig was then removed from the arena and placed back into their home cages. During the test phase, the pig was then reintroduced (individually) into the arena in which one of the two objects they were exposed to during the exploration phase had been replaced with a new one. [23]. Both the testing arena and the objects were cleaned with hot water and 70% alcohol between each separate test phase to minimise olfactory traces. Three measures of discrimination behaviour were calculated [22]. The first measure (D1) was the difference in exploration time for novel versus familiar objects, that is, the exploration time devoted to the novel object (TN) minus the time devoted to the familiar object (TF) (D1 = TN-TF). The second measure (D2 –*discrimination index*) used the difference in exploration time (i.e., D1), but then divided D1 by the total amount of exploration given to both the novel and familiar objects (D2 = (TN-TF)/(TN+TF)). The resulting D2 index can vary between +1 and −1, with a positive ratio showing a preference for novel objects and a ratio of zero corresponding to no preference [25]. The third measure was the recognition index (RI), i.e., the time spent investigating the novel object relative to the total time spent for object investigation (RI = TN/(TN + TF)), which is the main parameter of retention [26].

To monitor locomotor function, exploration activity and social interaction capacity the *olfactory discrimination of conspecifics scent (ODCST)* (adapted from [27]) was tested. Two stimuli were placed inside the arena, a familiar stimulus- a transparent plastic jar filled with straw bedding from the sow’s pen, and an unfamiliar stimulus- an identical empty plastic jar, was used. The testing arena was cleaned with hot water and 70% alcohol between each separate test phase to minimise olfactory traces. Each test lasted 5 min and the pigs' behaviour was recorded, including the accumulated time spent with and the number of visits made to the familiar and the unfamiliar stimuli was calculated [27]. The difference in exploration time for familiar versus unfamiliar stimuli (DF), that is, the exploration time devoted to the familiar stimulus (TF) minus the time devoted to the unfamiliar stimulus (TU), (DF = TF-TU), was also calculated.

### Blood, CSF, tissue sampling and euthanasia

Blood was collected from the umbilical vein at the start of the expermiments and from the jugular vein at end of the experiments, by venipuncture, into ice-chilled tubes coated with lithium heparin (BD Vacutainer^®^, 367884, Becton, Dickinson and Company (BD Medical), Franklin Lakes, New Jersey, USA) as an anticoagulant. The blood was then centrifuged at 3000 × g for 15 minutes, plasma was removed and frozen at -20°C until analysis.

CSF was collected by puncture of the *cysterna magna* at the end of the experiment [28]. Before sampling, the pigs were anesthetized using 0.5–1.5% air mixture of Fluothane (Zeneca, Gothenburg, Sweden) and O_2_ as a carrier gas, at approximately 0.5–1 L/min in a close-circuit respiratory system (Komesaroff Medical Developments, Melbourne, Australia). CSF samples from the pigs were collected directly into polypropylene tubes and frozen at -20°C until further analysis.

Piglets from the newborn unsuckled group were euthanized within 1 h after birth. Piglets from other experimental groups were euthanized immediately after CSF sampling, using a single dose of *i*.*v*. injected sodium pentobarbiturate (100 mg/kg b.wt.). The piglets’ brains were quickly dissected out and the hippocampi from the right hemisphere (n = 6 per group) were isolated and immediately frozen for further biochemical analysis, while the hippocampi from the remaining hemisphere (left) were dissected and immediately fixated for immunohistochemical analysis.

### Analyses of total protein and IgG

Total protein concentration (μg/ml) in the plasma and CSF samples, as well as in the serum, colostrum and the IG preparation was estimated according to Lowry et al [29], using bovine serum albumin (BSA, Sigma Cat#A5470, Sigma-Aldrich, St. Louis, MO, USA) as the standard.

The concentration of swine and bovine IgG (μg/ml) in the plasma and CSF samples, as well as in the swine serum, bovine and swine colostrum and the porcine IG preparation was analysed by single radial immunodiffusion [30], using anti-porcine IgG produced in rabbits (Sigma Cat#P0916) and purified porcine IgG as the standard (Sigma Cat#I4381); anti-bovine IgG produced in rabbits (Sigma Cat#B5645) and purified bovine IgG as the standard (Sigma Cat#I5506, all Sigma-Aldrich, St. Louis, MO, USA), respectively.

### ELISA of neurospecific proteins

The levels of microtubule associated protein 2 (MAP-2), doublecortin (DCX) and growth associated protein 43 (GAP 43) in homogenates of the hippocampal tissue samples were measured using the respective ELISA kits (Cat#MBS740491; Cat#MBS9350052; Cat#MBS017812). Tissue sample preparation and analysis were carried out according to the recommendations of the manufacturer (MyBiosource, Inc., San Diego, CA, USA). Briefly, the hippocampi were weighed before homogenization, then minced into small pieces and homogenized in 0.02 M PBS with a glass homogenizer, on ice (the buffer volume was approximately 1.5 times that of the tissue weight). The resulting suspension was subjected to two freeze-thaw cycles to further break down the cell membranes. After that, the homogenates were centrifuged for 15 minutes at 1500×g. The supernatant was removed and the assay was performed immediately. Since our tissue sample preparations were prepared in a 1:4 dilution, the recovery range for this dilution is between 93–107%, 92–104% and 92–104% for MAP2, GAP43 and DCX, respectively. The assay sensitivity is 0.1 ng/ml. Optical density was measured using a Spectra Max i3x Multi-Mode detection platform (Molecular Devices, LLC, Sunnyvale, CA, USA).

### Immunohistochemical analysis of the synaptic proteins

The coronal sections of the hippocampus, fixed with 4% formaldehyde in 0.1 M phosphate buffer (pH 7.38), were cut into 40 μm thick sections using a cryostat microtome (Jung CM 1500, Jung 2010 C, Leica, Germany). The immunohistochemical analysis was performed on free floating hippocampal sections. The sections were rinsed with PBS, permeabilized with 1% Tween-20 in PBS and treated with blocking solution (1% normal goat serum and 0.3% Triton X-100). Double immunofluorescence staining was performed. The presynaptic terminal marker protein, synaptophysin, was analysed using mouse monoclonal antibodies (Abcam Cat#ab8049, Abcam, Cambridge, MA, USA) in a 1:10 dilution, while rabbit polyclonal antibodies to synaptopodin (Abcam Cat#ab101883, Abcam, Cambridge, MA, USA) in a 1:1000 dilution were used to identify postsynaptic densities and dendritic spines. The hippocampal slices were incubated with the respective primary antibodies overnight at +4°C. AlexaFluor^®^ 488-conjugated donkey anti-mouse IgG and AlexaFluor^®^ 647-conjugated goat anti-rabbit IgG (Abcam Cat#ab150106 and Cat#ab150079, Abcam, Cambridge, MA, USA), respectively, were used as the secondary antibodies, and the slides were incubated for 1,5 h at room temperature. The hippocampal slices were then rinsed, placed on histological slides and mounted with Fluorescence Mounting Media (Dako, Glostrup, Denmark). The images of hippocampal tissue were analyzed using a confocal microscope (LSM 5 PASCAL, Zeiss, Germany). The amounts of synaptophysin-postitive and synaptopodin-positive individual puncta were counted for the entire hippocampal section in 10 randomly selected coronal sections per animal; estimation of the amount of synaptopodin and synaptophysin +/+ positive punctae as well as analysis of their colocalization on the images obtained was carried out using AxioVision Release, version 4.3 (Zeiss, Germany) in equal squares of the hippocampal sections (4624 μm^2^ test area). The colocalization coefficients M1 and M2, which describe the contribution of each one of the selected channels to the region of interest [31] were used to estimate the colocalization.

### Statistical analysis

Data are expressed as mean ± standard deviation (±SD). Ordinal, logistic regression was performed (JMP 13 Software, SAS Institute Inc., NC, USA) to assess the possible effects of sow and sex on the parameters measured. There was no significant effect of either sex or sow on any of the parameters measured; therefore, the male and female data, as well as the data from the different sow litters were combined for calculation of the means. Distribution of the parameters was assessed with the Kolmogorov-Smirnov normality test. A one-way ANOVA, followed by a Tukey post-hoc test was used to assess statistical differences between groups. For behavioural data analysis, a one sample t-test was used to compare D1, D2 and DF to 0.0 and the recognition index to 0.5, to determine if piglets displayed memory/recognition of the objects. These analyses were carried out using GraphPad Prism, version 7.0, (GraphPad Software, Inc., La Jolla, CA, USA). In all statistical analyses p<0.05 was considered significant.

## Results

### Immunoglobulin levels in blood plasma and cerebrospinal fluid

Significantly increased concentrations of porcine IgG (>10 mg/ml) were observed in the blood plasma of piglets fed swine colostrum (SC group), after the first 2 days of life, compared to that observed in the newborn unsuckled piglets (<0,1 mg/ml) (Table 2). Piglets fed infant formula only (IF group) or bovine colostrum (BC group) were IgG deficient. A relatively high level of blood plasma porcine IgG was observed in the groups i.v. infused with porcine IG. However, piglets injected with a high dose of swine immunoglobulins (IF+IGHD) revealed significantly (p<0.05) increased levels of IgG in blood plasma compared to the piglets injected with a low dose of swine immunoglobulins (IF+IGLD) (Table 2).

**Table 2:** The level of porcine and bovine IgG in blood plasma and cerebrospinal fluid (CSF) in piglets.

Plasma IgG concentration (μg/ml)						
	NB	IF	BC	IF+IGLD	IF+IGHD	SC
Porcine	86.9±45.3^a^	75.8±24.3^a^	92.9±20.2^a^	1777.3±1074.2^b^	6557.4±1299.1^c^	9772.1±543.9^d^
Bovine	n/a	n/a	6326.1±860.5	n/a	n/a	n/a
CSF IgG concentration (μg/ml)						
	NB	IF	BC	IF+IGLD	IF+IGHD	SC
Porcine	3.8±0.8^a^	4.1±1.1^a^	3.4±0.7^a^	16.1±3.1^b^	86.7±19.4^c^	176.0±10.8^d^
Bovine	n/a	n/a	29.5±10.7	n/a	n/a	n/a

Unsuckled newborn piglets (NB, n = 6) and newborn piglets fed with either an infant formula (IF, n = 6), bovine colostrum (BC, n = 6), an infant formula + i.v. infusion of sow serum (IF+IGLD, n = 6), an infant formula + i.v. infusion of porcine immunoglobulins (IF+IGHD, n = 6), or swine colostrum (SC, n = 6). Data are presented as mean±SD. Small letters given with result bars describe significant differences within rows when p<0.05. N/a–non applicable.

Bovine IgG was present in the blood plasma of the piglets fed bovine colostrum (Table 2).

Regarding the IgG levels in the CSF, the data obtained in the present study showed that porcine IgG could cross the blood-brain barrier (BBB) and was found in the CSF (Table 2). Only trace amounts of IgG (<5 μg/ml) were observed in the CSF of the newborn piglets and in the experimental piglets with low plasma levels of IgG (IF and BC groups), while increasing the plasma IG level by i.v. infusion of porcine IG led to an increase in CSF IgG levels in a dose-dependent manner (group IF+IGHD revealed significantly (p<0.05) higher levels of IgG in CSF compared to the IF+IGLD group). At the same time, feeding sow colostrum to the piglets by oral gavage (SC group) led to a significant increase in CSF IgG levels (up to 200 μg/ml).

The bovine IgG from the bovine colostrum was also able to cross the BBB and was found in the CSF of the piglets (Table 2).

### Neurospecific proteins in the hippocampus

The levels of neurospecific proteins, GAP43, DCX and MAP2 were measured in the hippocampal homogenates (Table 3). GAP43 levels in the hippocampal tissue of piglets fed either formula (IF) or bovine colostrum (BC), during the first 48 hours of life, were significantly (p<0.05) lower than those observed in the newborn, unsuckled piglets (NB group). Intravenous infusion of swine serum (low dose of porcine immunoglobulins), as well as the feeding of swine colostrum to piglets (IF+IGLD and SC groups), prevented the decrease in hippocampal GAP43, maintaining levels similar to those observed at birth. Intravenous infusion of purified porcine IG at a high dose (IF+IGHD group) significantly (p<0.05) increased the level of GAP43, 48 hours following birth (Table 3).

**Table 3:** The levels of neurospecific proteins, GAP43, DCX and MAP2 in the hippocampal tissue of piglets.

Concentration, ng/mg tissue						
	NB	IF	BC	IF+IGLD	IF+IGHD	SC
GAP43	3.85±0.20^a^	0.60±0.44^b^	0.02±0.01^c^	3.45±0.41^a^	4.51±0.33^d^	3.56±0.41^a^
DCX	1.27±0.59^a^	0.47±0.33^b^	1.05±0.25^a^	1.42±0.48^a^	2.55±0.53^c^	2.88±0.76^c^
MAP2	7.26±2.65^a^	7.66±1.06^a^	27.40±13.49^b^	46.08±11.46^bc^	68.98±0.55^d^	49.42±6.73^c^

Unsuckled newborn piglets (NB, n = 6) and newborn piglets fed with either an infant formula (IF, n = 6), bovine colostrum (BC, n = 6), an infant formula + i.v. infusion of sow serum (IF+IGLD, n = 6), an infant formula + i.v. infusion of porcine immunoglobulins (IF+IGHD, n = 6), or swine colostrum (SC, n = 6). Data are presented as mean±SD. Small letters given with result bars describe significant differences within rows when p<0.05. N/a–non applicable.

A low DCX level was observed in the hippocampal tissue of the newborn, unsuckled pigs (NB group). The intravenous infusion of a low dose of porcine IG (IF+IGLD group), as well as bovine colostrum feeding (BC group) maintained the levels of DCX at the levels observed directly after birth. Infant formula feeding (IF group) led to a significant (p<0.05) decrease in hippocampal DCX, 48 hours following birth. Feeding piglets with swine colostrum (SC group) led to a significant increase (p<0.05) in DCX levels, 48 hours post birth. A similar effect was also observed following the intravenous infusion with a high dose of porcine IG (IF+IGHD group) (Table 3).

The lowest MAP2 level was observed in the hippocampal tissue of the newborn, unsuckled piglets (NB group) and in the piglets fed with infant formula (IF group). Bovine colostrum feeding (BC group) and the intravenous infusion of a low dose of porcine IG (IF+IGLD group) led to significantly higher (p<0.05) MAP2 levels, 48 hours following birth. The piglets fed with swine colostrum (SC group) displayed significantly (p<0.05) increased MAP2 levels, 48 hours following birth. Nevertheless, the intravenous infusion of a high dose of purified swine IG (IF+IGHD group) resulted in an even more pronounced increase in MAP2 levels at the end of the study (Table 3).

### Immunohistochemical investigation of synaptic proteins

**Fig 1:**
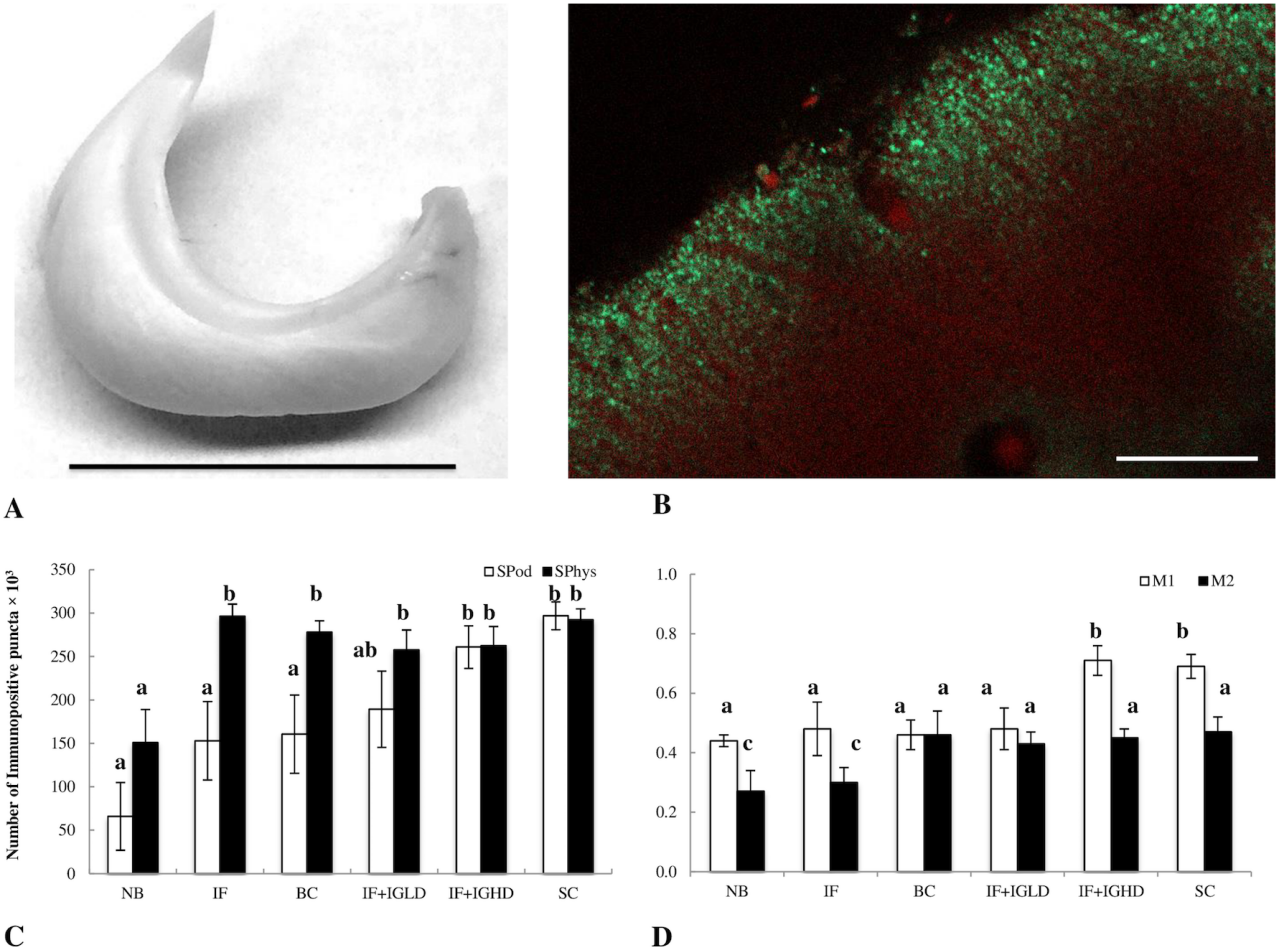
**A) The left hippocampus of the piglet after dissection. Scale bar– 1.5 cm. B) Immunostaining of the subiculum of piglets. Synaptopodin- positive punctae (red) and synaptophysin- positive punctae (green). Scale bar– 100 μm. C) The density of stained regions for the synaptic proteins, synaptophysin (SPhys) and synaptopodin (SPod), in the hippocampus of piglets as analysed with immunohistochemistry. D)The colocalisation coefficients M1 and M2 of synaptophysin-positive and synaptopodin-positive puncta in the hippocampus of piglets as analysed with immunohistochemistry.** Unsuckled newborn piglets (NB, n = 6) and newborn piglets fed with either an infant formula (IF, n = 6), bovine colostrum (BC, n = 6), an infant formula + i.v. infusion of sow serum (IF+IGLD, n = 6), an infant formula + i.v. infusion of porcine immunoglobulins (IF+IGHD, n = 6), or swine colostrum (SC, n = 6). Data are presented as mean±SD. Small letters given with result bars describe significant differences when p<0.05.

Since no significant differences in the synaptophysin- and synaptopodin- positive punctae distribution were observed between CA1, CA3 areas, dentate gyrus and the subiculum in piglets’ hippocampi, the values obtained from all the mentioned regions were combined for calculation. A 50% increase in the density of synaptophysin-positive punctae, 48 hours following birth, was observed in the IF, BC, IF+IGLD, IF+IGHD and SC groups, compared to that of the NB group. At the same time, a significantly increased (p<0.05) amount of synaptopodin-positive puncta were observed in piglets from the IF+IGHD and SC groups, but not in the IF, BC and IF+IGLD groups, compared to that observed in the NB group. Interestingly, the IF+IGHD and SC groups displayed significantly increased colocalization coefficient M1 values (around 0.7) in comparison to those obtained in the NB, IF, BC and IF+IGLD groups (around 0.47). The colocalization coefficient M2 in the NB and IF groups was approximately 0.3, while in piglets from the BC, IF+IGLD, IF+IGHD and SC groups, it was approximately 0.45 (Fig 1).

### Piglets’ behaviour

Parameters observed in the piglets fed with swine colostrum (Group SC) were considered as physiological and representative for piglets 48 hours following birth.

The results of the NOR test demonstrated a lower exploratory activity and impaired short-term memory abilities in piglets that were completely deprived of immunoglobulins (IF and BC groups). Piglets in the IF group spent less time exploring the novel object (D1<0) and piglets from the BC group demonstrated a very low difference in exploration time between familiar and unfamiliar objects (D1). Both the IF and BC groups revealed a negative discrimination index (D2). However, the D2 values in both IF and BC groups didn't differ significantly from 0.0, corresponding to no preference. The recognition index (R1) in these two groups was not significantly different from 0.5, which indicates impaired retention. Piglets, which received porcine IG supplementation, i.v. or orally, (IF+IGLD, IF+IGHD and SC groups) spent significantly (p<0.05) more time exploring the unfamiliar object in the test phase (D1>0), in comparison to the IF and BC piglet groups. The discrimination index D2 in group IF+IGLD was positive, but didn’t differ significantly (p>0.05) from 0.0, indicating no preference. The D2 values in both IF+IGHD and SC groups were positive, and significantly (p<0.05) differed from 0.0, indicating explorative activity directed towards the novel object. The recognition index R1 in piglets that received porcine IG supplementation, i.v. or orally (IF+IGLD, IF+IGHD and SC groups), was significantly (p<0.05) different from 0.5, showing a high retention level (Table 4).

**Table 4:** Explorative activity and olfactory discrimination tested in the novel object recognition (NOR) and olfactory discrimination of conspecifics scent (ODCST) tests, performed in piglets.

	IF	BC	IF+IGLD	IF+IGHD	SC
NOR *(Novel Object Recognition)*					
D1	-26.0±12.70^a^*	5.0±2.31^b^*	19.0±15.59^c^*	28.5±5.20^d^*	35.5±6.35^e^*
D2	-0.5±0.33^a^	-0.2±0.53^a^	0.3±0.26^ab^	0.5±0.07^b^**	0.4±0.18^b^*
RI	0.3±0.17^a^	0.4±0.23^a^	0.7±0.13^b^	0.8±0.03^b^*	0.7±0.09^b^*
ODCST *(Olfactory discrimination of conspecifics scent)*					
DF	9.67±6.87^a^*	-2.00±4.73^a^	6.00±3.28^a^*	63.33±16.93^b^*	71.50±34.51^ab^*

Unsuckled newborn piglets fed with either an infant formula (IF, n = 6), bovine colostrum (BC, n = 6), an infant formula + i.v. infusion of sow serum (IF+IGLD, n = 6), an infant formula + i.v. infusion of porcine immunoglobulins (IF+IGHD, n = 6), or swine colostrum (SC, n = 6). D1, the difference in exploration time for novel versus familiar objects; D2, discrimination index; RI, recognition index; DF, the difference in exploration time for familiar “pig” versus unfamiliar stimuli (for ODCST the difference in exploration time). Data are presented as mean±SD.

Small letters given with result bars describe significant differences within rows when p<0.05.

* describes significant (p<0.05) differences between actual value and hypothetical value (HP). HP for D1 and D2 = 0.00; HP for RI = 0.50.

Measuring the olfactory discrimination between objects with the ODCST showed that all piglets from the IF, IF+IGLD, IF+IGHD and SC groups visited the familiar “pig” stimulus frequently and spent significantly (p<0.05) more time near the familiar stimulus in comparison to the unfamiliar one (D1>0). However, piglets in the IF, BC and IF+IGLD groups, spent less time near the familiar “pig” stimulus than piglets in the IF+IGHD and SC groups. Moreover, the piglets in the BC group showed no discrimination ability (DF was not significantly different from 0.0, Table 4).

## Discussion

The newborn piglet is generally accepted as a relevant model for human neonates and it is likely that similar factors are involved in the functional maturation of the brain in human newborns. The pig model allows us to assess both structural and functional features of brain development and their regulation by external factors. Moreover, use of the unsuckled neonatal pig model, which is hypogammaglobulinemic at birth, provides us with a possibility to examine the dose-dependency, as well as the species-specificity of immunoglobulin transfer effects on brain development. Thus, in the present study we made use of swine IG in different doses to assess the role of maternal IG transfer in early postnatal development. Moreover, we also tested bovine colostrum which is known to improve intestinal, digestive and immune functions in preterm pigs [32] and is currently being investigated in a clinical pilot trial [33] as the first enteral feeding for preterm infants, to compare the ability of foreign IG to cross the BBB and affect brain development. In the current study, the assessment of functional and structural changes in the hippocampus of the piglets was carried out by investigation of synaptic and neuronal plasticity-associated proteins, as well as measurements of changes in piglet behaviour.

A previous study by our lab showed a positive relationship between plasma levels of IG and hippocampus development during the first 3 days of postnatal development [16]. The hippocampus is involved in memory and learning, which are processes shown to be deficient in premature infants. Thus, altered hippocampal development involving synaptic and neuronal plasticity may represent an etiology by which memory and learning deficits manifest in preterm children, later on in life. Moreover, hippocampal subareas are among the least genetically programmed brain regions and are thus more vulnerable to environmental influence [34].

In the present study a deficiency of swine IgG was observed in piglets from the NB, IF and BC groups (totally deprived of swine IG). The highest plasma levels of swine IgG were observed in piglets from the IF+IGHD and SC groups. Plasma levels of bovine IgG in piglets from the BC group were high (similar to the level of porcine IgG observed in the IF+IGHD group, Table 2). The high plasma levels of infused IgG were maintained for at least 48 hours after infusion.

Regarding the transfer of IG from the circulation to the CSF, the present data demonstrated passage of both species-specific (swine) and foreign (bovine) IG via the BBB in the newborn pigs. This finding, however, contradicts data obtained by Harada et al. [35], which showed that the permeability of the BBB in newborn piglets was exclusive to species-specific IG. Moreover, the transfer of species-specific IG via the BBB seems to be dose-dependent in piglets, as the piglets which had the highest levels of IgG in circulation also had the highest levels of IgG in the CSF (IF+IGHD and SC groups).

To estimate neuronal development, migration and maturation, neuronal plasticity-associated protein content of the hippocampal tissue from the piglets was analyzed.

GAP43 is a protein expressed at high levels in neuronal growth cones during early development [36] and is considered to be a crucial component of the axon and presynaptic terminal. GAP43 plays a role in axonal pathfinding [37], neurotransmitter release [38], and synaptic plasticity [39]. In the current study a decrease in hippocampal levels of GAP43 was observed in piglets deprived of swine immunoglobulins, while infusion of swine serum or swine colostrum feeding were able to maintain the level of GAP43 similar to that observed in newborn pigs. An intravenous infusion of purified swine IG not only maintained GAP43 levels, but also stimulated an increase in GAP43, postnataly. Bovine colostrum feeding was not effective in maintaining the GAP43 levels at the initial levels observed in the newborn piglets.

DCX is the marker protein associated with neuronal differentiation and cellular migration and maturation, including synaptogenesis due to the nearly exclusive expression of DCX in developing neurons [40]. In the present study we found that the newborn piglets had the lowest level of DCX in hippocampal tissue. Infusion of swine serum containing a low dose of swine IG (IF+IGLD group) as well as bovine colostrum feeding (BC group), maintained an unchanged DCX level, while artificial feeding with infant formula(IF group) led to a decrease in the levels of DCX in the hippocampus. At the same time, feeding piglets with sow colostrum (SC group) or infusing piglets with a high dose of purified swine IG (IF+IGHD group) led to a significant increase in DCX levels, 48 hours post birth.

A similar pattern was observed with regards to MAP2, with the lowest protein level observed in the newborn piglets, as well as in those deprived of species-specific IG in a sufficient dose (IF and BC groups). MAP2 is known to associate with microtubules to provide support to the neuronal cytoskeleton and is important for dendritic structural plasticity control and neurite growth [41]. In addition, MAP2 is essential for cellular functions such as the integration of synaptic inputs, local signal transduction, protein trafficking and synaptic plasticity [42,43]. In both human and experimental models of epilepsy, decreases in cortical and hippocampal MAP2 immunoreactivity, along with MAP2 dephosphorylation, have been reported [44,45]. The intravenous infusion of swine IG at a low dose (IF+IGLD group) or swine colostrum feeding (SC group) led to an increase in the hippocampal MAP2 level, while the intravenous infusion of swine IG at a high dose (IF+IGHD group) produced an even more pronounced effect.

In the current study, we also documented that the amount of synaptophysin-postitive and synaptopodin-positive punctae also appear to be dependent on IG supplementation. It is worth mentioning that data on the distribution of synaptic proteins in pigs’ hippocampi is still lacking. It is known that in guinea pigs the distribution of synaptic proteins, such as synaptophysin and synaptopodin, differs between hippocampal regions [46]. In human infants the synaptophysin is equally distributed in CA1, CA3 and subiculum, while in rats its’ distribution differs significantly between all hippocampal regions [47]. We have observed no differences in synaptophysin and synaptopodin distribution between different hippocampal regions (CA1, CA3 areas, subiculum and dentate gyrus).

Synaptophysin is an integral glycoprotein localized to the membrane of presynaptic boutons and serves as a marker for presynaptic terminal development [48,49] and can be used for the quantification of synapses [50]. Synaptopodin, a specific marker of labile mushroom spines, plays an important role in neuronal network plasticity [51] and has been used as a marker of synaptic maturation [52]. Development of neuronal plasticity to a great extent depends on formation of labile mushroom-shaped dendritic spines which are well-known to be capable of rapid changes in form and size, resulting in modulation of synaptic transmission. Synaptopodin is especially important for remodeling of the spine cytoskeleton and neuronal network plasticity [53]. There is a correlation between memory and the density and plasticity of the labile spines in the cortical areas of the brain [50]. The current study demonstrated a 50% increase in the density of synaptophysin-positive punctae, as well as in the colocalization coefficient M2, following 48 hours of postnatal development, for all piglet groups compared to that observed in the newborn pigs. However, the density of synaptopodin-positive punctae and the colocolization of synaptopodin with synaptophysin (colocalization coefficient M1) seems to be dependent on immunoglobulin supplementation, as it was only significantly increased in the piglets that were either intravenously infused with swine IG at a high dose (IF+IGHD group) or fed with sow colostrum (SC group). So, the number of synaptopodin-containing mature dendritic spines, and consequently mature synaptic connections, is dependent on sufficient IG supplementation. The decreased amount of synatopodin-positive labile spines might underlie changes of neuronal network plasticity [54] and testify to the cognitive impairments characteristic of premature infants. The data obtained from behavioural studies demonstrated that piglets that were totally deprived of species-specific IG (IF and BC groups) demonstrated decreased exploratory activity in comparison to the piglets that received swine IG (IF+IGLD, IF+IGHD and SC groups). Moreover, the NOR test revealed an impairment in the short-term memory of piglets that were deprived of species-specific immunoglobulins, impaired retention (RI doesn’t differ from 0.5) and a discrimination index value that doesn’t differ significantly from 0.0 (D2≤0), which corresponds to no preference towards any object (IF and BC groups). Pigs have a rather advanced social cognition [55], where the olfactory discrimination plays one of the main roles [27]. ODCST results revealed that the majority of the piglets (except those from the BC group) demonstrated olfactory discrimination. Piglets deprived of swine immunoglobulins (IF and BC groups) visited familiar object less frequently and spent less time nearby when compared to piglets receiving species-specific IG supplementation, despite the stimuli discrimination.

Taken together data concerning neuronal plasticity, content of synaptic-associated proteins and piglets’ behaviour allows us to suggest that foreign (bovine) IG, despite their ability to pass BBB, cannot ensure proper neuronal pathfinding, migration and synaptic maturation, as well as the development of species-specific behavioral patterns (unlike species-specific porcine IG). Thus, the already known beneficial effects of IG intravenous supplementation are possibly due not only to their immunological effects, but also to some “extra-immunological” properties of IG.

The majority of the effects observed following the intravenous infusion of immunoglobulins are due to the their immunological properties. There are however beneficial effects which have been described (not on brain development) following intravenous infusion of immunoglobulins; e.g. in the treatment of epilepsy (an anticonvulsant effect and significantly increased seizure threshold) both in patients and in animal models [56,57]. Authors suggest that the evidence for the non-immunological actions of immunoglobulins is related to the time course of the response to treatment of patients with different forms of epilepsy (an immediate response suggests a direct neuromodulating effect) [57]. The possible mechanisms of extra-immunological effects of immunoglobulins have not yet been elucidated and require further investigation, especially with regards to the development of the CNS. In fact, only one publication from our lab [16] indirectly points out the effects of absorbed immunoglobulins on brain function/development.

In summary our data on the piglets’ behaviour, neuronal development and synaptic plasticity allows us to suggest that social discrimination and object-directed explorative activity in piglets, as well as neuronal maturation and synaptic development are dependent on a sufficient amount of species-specific immunoglobulins in the circulation. We have mentioned above that bovine colostrum is currently being investigated in a pilot clinical trial as the first enteral feeding for preterm infants [33]. Moreover, we suspect that immunoglobulins from bovine colostrum could improve the general immune status but would not be sufficient to improve the neurological development in preterm, and especially extremely preterm, infants. The intravenous immunoglobulins infusion is used in clinical practice as treatment for sepsis and infections development in preterm infants [58,59], however, its’ possible neurodevelopmental benefits are underestimated. The positive effects of intravenous infusion of species-specific immunoglobulins on behaviour and neuronal plasticity observed in the present newborn unsuckled piglet model might be relevant with respect to the clinical use of immunoglobulins in preterm infants to ensure adequate brain development and protect such infants against the development of cognitive disorders in the future.
